# Delivery of cytoplasmic and apoplastic effectors from *Phytophthora infestans* haustoria by distinct secretion pathways

**DOI:** 10.1111/nph.14696

**Published:** 2017-07-31

**Authors:** Shumei Wang, Petra C. Boevink, Lydia Welsh, Ruofang Zhang, Stephen C. Whisson, Paul R. J. Birch

**Affiliations:** ^1^ Division of Plant Sciences University of Dundee (at JHI) Errol Road Invergowrie Dundee DD2 5DA UK; ^2^ Cell and Molecular Sciences James Hutton Institute Errol Road Invergowrie Dundee DD2 5DA UK; ^3^ Potato Engineering and Technology Research Centre of Inner Mongolia University West College Road 235 Hohhot 010021 China

**Keywords:** apoplastic effector, effector translocation, effector‐triggered susceptibility, plant disease, plant immunity, secretion, virulence

## Abstract

- The potato blight pathogen *Phytophthora infestans* secretes effector proteins that are delivered inside (cytoplasmic) or can act outside (apoplastic) plant cells to neutralize host immunity. Little is known about how and where effectors are secreted during infection, yet such knowledge is essential to understand and combat crop disease.
- We used transient *Agrobacterium tumefaciens*‐mediated *in planta* expression, transformation of *P. infestans* with fluorescent protein fusions and confocal microscopy to investigate delivery of effectors to plant cells during infection.
- The cytoplasmic effector Pi04314, expressed as a monomeric red fluorescent protein (mRFP) fusion protein with a signal peptide to secrete it from plant cells, did not passively re‐enter the cells upon secretion. However, Pi04314‐mRFP expressed in *P. infestans* was translocated from haustoria, which form intimate interactions with plant cells, to accumulate at its sites of action in the host nucleus. The well‐characterized apoplastic effector EPIC1, a cysteine protease inhibitor, was also secreted from haustoria. EPIC1 secretion was inhibited by brefeldin A (BFA), demonstrating that it is delivered by conventional Golgi‐mediated secretion. By contrast, Pi04314 secretion was insensitive to BFA treatment, indicating that the cytoplasmic effector follows an alternative route for delivery into plant cells.
- *Phytophthora infestans* haustoria are thus sites for delivery of both apoplastic and cytoplasmic effectors during infection, following distinct secretion pathways.

## Abstract 2

See also the Commentary on this article by Van den Ackerveken, **216**: 8–10.

## Introduction

Successful plant pathogens secrete effector proteins that act outside (termed apoplastic effectors) or inside (cytoplasmic effectors) plant cells to suppress or otherwise manipulate host processes (Asai & Shirasu, 2015). Bacterial plant pathogens use a variety of secretion systems to deliver effectors. Among these are the well‐characterized type II secretion system, which delivers apoplastic effectors and plant cell wall‐degrading enzymes, and the type III secretion system (T3SS), which is an elaborate mechanism for delivering effector proteins into the cytoplasm of plant cells (Alfano & Collmer, 2004; Pfeilmeier *et al*., 2016). Filamentous (fungal and oomycete) plant pathogens also secrete effectors that act inside or outside of host cells. However, in contrast to bacterial pathogens, the delivery of cytoplasmic effectors from filamentous pathogens into plant cells has rarely been directly visualized and is poorly understood.

Fungal effector research has historically been driven by the search for avirulence proteins, which are detected by matching plant resistance proteins. Such effectors include the *Cladosporium fulvum* Avirulence 2 (Avr2), Avr4 and Avr9 proteins, which are predicted apoplastic effectors that are detected by cell surface *C. fulvum* (Cf) resistance proteins (de Wit, 2016). By contrast, avirulences characterized in the rice blast pathogen *Magnaporthe oryzae* (Jia *et al*., 2000) and the flax rust pathogen *Melampsora lini* (Dodds *et al*., 2004) are generally cytoplasmic effectors detected by plant nucleotide‐binding leucine‐rich repeat (NB‐LRR) resistance proteins. Although avirulence proteins have yet to be characterized in the maize (*Zea mays*) pathogen *Ustilago maydis*, this fungus also secretes both apoplastic (e.g. Mueller *et al*., 2013) and cytoplasmic (e.g. Tanaka *et al*., 2014) effectors to suppress plant defences.

Direct delivery of cytoplasmic fungal effectors to the inside of plant cells has only been visualized by live‐cell imaging in the case of *M. oryzae. M. oryzae* forms specialized invasive hyphae (IH) that occupy living host cells. Apoplastic effectors are secreted into the extra‐invasive hyphal membrane (EIHM) compartment, surrounding the IH, but do not enter the plant cytosol. By contrast, cytoplasmic effectors accumulate at a membrane‐rich structure called the biotrophic interfacial complex (BIC), before being translocated across the EIHM to the cytoplasm of living rice cells (Khang *et al*., 2010). It has been shown that, whereas the apoplastic *M. oryzae* effector biotrophy‐associated secreted protein 4 (Bas4) was conventionally secreted from the IH to the EIHM compartment, the cytoplasmic effector Pwl2 (for Pathogenicity toward Weeping Lovegrass) was delivered into the host cell from the BIC by nonconventional secretion (Giraldo *et al*., 2013).

Although plant pathogenic oomycetes share morphological and developmental traits with fungi, they are nevertheless evolutionarily unrelated; similarities in behaviour and appearance are therefore often regarded to be the products of convergent evolution (Latijnhouwers *et al.,* 2004). One of the best characterized oomycetes is *Phytophthora infestans*, the cause of late blight disease, which is a major global threat to potato (*Solanum tuberosum*) and tomato (*Solanum lycopersicum*) production (Fry *et al*., 2015; Kamoun *et al*., 2015). *Phytophthora infestans* secretes both apoplastic and cytoplasmic effectors (Kamoun, 2006; Hein *et al*., 2009; Whisson *et al*., 2016). Among the best characterized *P. infestans* apoplastic effectors is EPIC1, which targets defence‐associated host proteases in the plant extracellular space (Tian *et al*., 2007; Song *et al*., 2009; Kaschani *et al*., 2010; Dong *et al*., 2014). However, as with other oomycete apoplastic effectors, the site and mode of secretion of EPIC1 are unknown.

Cytoplasmic oomycete effectors include the RXLR class, containing the conserved Arg‐any amino acid‐Leu‐Arg (RXLR) peptide motif that is required for these proteins to be delivered into plant cells (Whisson *et al*., 2007, 2016; Dou *et al*., 2008; Anderson *et al*., 2015). The importance of this effector class is epitomized by gene‐for‐gene resistance to *P. infestans*, which is governed by recognition of specific RXLR effectors by host NB‐LRR resistance proteins inside plant cells (e.g. Hein *et al*., 2009; Anderson *et al*., 2015). Recent efforts have revealed the host proteins targeted by many RXLR effectors from pathogens such as *P. infestans* (Anderson *et al*., 2015; Whisson *et al*., 2016). RXLR effectors such as AVR3a (Whisson *et al*., 2007, 2016), AVR2 (Gilroy *et al*., 2011) and Avr1b (Liu *et al*., 2014) accumulate at haustoria, implicating this structure as their site of secretion. However, the mode of secretion is unknown and, to date, delivery of an RXLR effector to the inside of a host cell has yet to be directly observed (Whisson *et al*., 2016).

Here, we describe a study of secretion and delivery, during infection, of two effectors from *P. infestans*: the cytoplasmic effector Pi04314 (Boevink *et al*., 2016) and the apoplastic effector EPIC1. Previous attempts to visualize translocation of the RXLR effector AVR3a as a monomeric red fluorescent protein (mRFP) fusion protein using confocal microscopy were unsuccessful, potentially as a consequence of the fact that AVR3a is dispersed and diluted in the host cytoplasm (Whisson *et al*., 2007, 2016). We thus selected Pi04314 as an RXLR effector with a defined activity that accumulates at a distinct site in the plant cell. Pi04314 accumulates in the plant nucleoplasm and nucleolus, where it interacts with protein phosphatase 1 catalytic (PP1c) subunits via an R/KVxF motif, causing their re‐localization from the nucleolus to the nucleoplasm (Boevink *et al*., 2016). Pi04314 acts as a regulatory subunit to form PP1c holoenzymes, presumably to dephosphorylate target host proteins to promote late blight disease. Using confocal microscopy, we investigated where the effectors Pi04314 and EPIC1 are secreted during infection, and whether detection of the former can be observed in the host nucleus, its site of action. Using brefeldin A (BFA), a well‐characterized inhibitor of Golgi‐mediated secretion that has been used to study *M. oryzae* effector secretion (Giraldo *et al*., 2013), we investigated whether Pi04314 and EPIC1 are conventionally secreted by the pathogen during infection.

## Materials and Methods

### *Phytophthora infestans* culture and *Nicotiana benthamiana* inoculation

*Phytophthora infestans* wild‐type strains 88069 and 3928A and transgenic lines were cultured as described by Grenville‐Briggs *et al*. (2005). Infection inoculum was prepared and inoculated in 10‐μl droplets onto wounded plant leaves as described by Whisson *et al*. (2007, 2016). Wild‐type *Nicotiana benthamiana* and transgenic lines with the plasma membrane tagged with the GFP‐Lti6, a low temperature induced protein tagged green fluorescent protein fusion (Kurup *et al*., 2005), and the nucleus labelled with CFP‐*Nb*H2BN histone H2B tagged cyan fluorescent protein (Goodin *et al*., 2007), were used for *P. infestans* infection.

### Vector construction and *Agrobacterium tumefaciens* transient assays

*Phytophthora infestans* RXLR effector gene Pi04314 (Boevink *et al*., 2016) was cloned with and without the sequence encoding the predicted signal peptide. Gene‐specific primers (Supporting Information Table S1) modified to contain restriction enzyme recognition sites were used in polymerase chain reactions (PCRs) to amplify the gene from genomic DNA of isolate 88069. The KVTF mutant motif was generated using a mismatch reverse primer, where KVTF was replaced by alanines. PCR products were purified and digested with *Cla*I and *Asi*SI restriction endonucleases and ligated into the same restriction sites in the plasmid pPL‐mRFP to yield effector‐mRFP C‐terminal fusions (Avrova *et al*., 2008). The fusion sequences were PCR amplified with primers including flanking gateway recombination sites at both terminal coding sequences using nested PCR (Table S1), and purified PCR products were recombined using Gateway cloning into pDONR201 (Invitrogen) to generate entry clones. The entry effector clones were recombined with pB2GW7 for expression of fusion proteins *in planta* (Karimi *et al*., 2007) and electroporated into *Agrobacterium tumefaciens* strain AGL1.

*Agrobacterium tumefaciens* transient transformation assays (ATTAs) were performed essentially as described by Kunjeti *et al*. (2016). Briefly, *A. tumefaciens* transformed with protein expression vectors were grown at 28°C overnight in yeast‐extract and beef (YEB) medium containing selective antibiotics. They were then pelleted by centrifugation and resuspended in infiltration buffer (10 mM 2‐(N‐morpholino)ethanesulfonic acid (MES), 10 mM MgCl_2_ and 200 mM acetosyringone, pH 7.5) to a final concentration, that is, optical density at 600 nm (OD_600_), of 0.1, and incubated at room temperature for at least 2 h before infiltration into leaves. *Nicotiana benthamiana* plants were grown in a controlled environment glasshouse at 22°C with 55% humidity and 16 h light d^−1^. Three middle leaves were selected from 4‐wk‐old *N. benthamiana* plants for agro‐infiltratration to express mRFP on one half of the midvein as a control, and an effector cloned into the same vector on the other half of the same leaf. After 1 d, each infiltration site was inoculated with 10 μl of zoospores (50 000 zoospores ml^−1^) from *P. infestans* isolate 88069. Lesion development was measured at 7 d post inoculation (dpi) (McLellan *et al*., 2013). Eighteen leaves from six individual plants were used for each of three replicates (*n *=* *108 per construct). Graphs present lesion development relative to the control, with error bars representing ± SE. A one‐way ANOVA test was conducted to identify statistically significant differences.

### *Phytophthora infestans* transformation vector construction and transformation

Full‐length (including signal peptide) EPIC1 (PITG_09169; EEY55256) Pi04314, and enhanced GFP (EGFP) were introduced into the oomycete expression vector pTOR, driven by the oomycete Ham34 promotor (Ham34P; Judelson *et al*., 1991), using standard methods for restriction endonuclease‐mediated cloning. The Ham34P‐gene fusions were PCR amplified with Ham34P and gene‐specific primers with 5′ restriction recognition sites. The Ham34P‐eGFP fusion was introduced first into a *Hin*dIII site in pPL‐mRFP (Avrova *et al*., 2008) to generate pPL‐RAG. Ham34P‐EPIC1 and Ham34P‐Pi04314 were then cloned ahead of mRFP in pPL‐RAG to yield Ham34P‐effector‐mRFP C‐terminal fusions. Transformation of *P. infestans* was achieved using a modified PEG‐CaCl_2_‐lipofectin protocol (Judelson *et al*., 1991), modified as described in Avrova *et al*. (2008). Transformed *P. infestans* lines were maintained in the dark at 19°C on rye agar containing 20 μg ml^−1^ geneticin antibiotic.

### Confocal microscopy

*Nicotiana benthamiana* leaf pieces were mounted on slides and imaged using a Nikon A1R confocal microscope and water dipping lenses. EGFP and chloroplast autofluorescence were imaged with 488 nm excitation and emissions collected between 500 and 530 nm, respectively. CFP was imaged with 456 nm excitation and emissions were collected between 500 and 530 nm. Imaging of mRFP was conducted using 561 nm excitation and emissions were collected between 600 and 630 nm. The pinhole was set to 1 Airy unit for the longest wavelength fluorophore. For localization of effector‐mRFP fusions expressed in the GFP‐LTi6b *N. benthamiana* transgenic line, and re‐localization of N‐terminal tagged GFP‐PP1c (Boevink *et al*., 2016) coexpressed with Pi04314 or Pi04314kvtf fusions in wild‐type *N. benthamiana*, leaf tissue was imaged 2 d after *A. tumefaciens* infiltration. Single optical section images and z‐series were collected from leaf cells expressing low levels of the fusion proteins to minimize the potential for artefacts associated with high levels of protein overexpression. For effector re‐entry, secretion and translocation assays, projections were collected from leaf tissue that was not heavily colonized by *P. infestans* to minimize the potential for interference from autofluorescence from the cell damage and death caused by *P. infestans*. For BFA treatment *in planta*, the stock solution of BFA is 5 mg ml^−1^ in dimethyl sulphoxide (DMSO). Leaf tissue infected by transformants was infiltrated with 50 μg ml^−1^ BFA (Boevink *et al*., 2011), and imaged after 3 h.

For fluorescence recovery after photobleaching (FRAP) experiments, haustoria surrounded by red fluorescence were chosen for bleaching. Photobleaching was performed using 488 nm emission at 80% laser power for 5 s. After three iterations of photobleaching, the fluorescence signal was attenuated significantly. Imaging before and after bleaching was conducted with 5% laser power. For quantitative analyses, red fluorescence covering entire haustoria was measured using the nis‐elements software package (Nikon) as the total fluorescence intensity of pixels within a region of interest (ROI) drawn to encompass the haustorium. Image processing for figures was conducted with Adobe photoshop cs2 and Adobe illustrator cs5.1.

### SDS‐PAGE and immunoblot analysis

*Phytophthora infestans* wild type and transformants were cultured in amended lima bean (ALB) liquid medium (Bruck *et al*., 1981). Mycelium was harvested by centrifugation at 6 dpi. To examine the effects of BFA on effector secretion *in vitro*, mycelia of transformants expressing either Pi04314‐mRFP or EPIC1‐mRFP were incubated in 1 ml of ALB liquid medium for 24 h post inoculation (hpi) with 50 μg ml^−1^ BFA. The culture filtrate (CF) was retained separately after filter sterilization, and four times the sample volume of cold (−20°C) acetone (Thermo Fisher Scientific, Loughborough, UK) was used to precipitate proteins overnight, and protein was precipitated at 10 000 ***g*** for 10 min. Leaf discs were harvested 2 d after agro‐infiltration (with agrobacteria at an OD_600_ of 0.5) to express Pi04314‐mRFP, with or without a signal peptide, and Pi04314kvtf‐mRFP. One‐centimetre‐square leaf discs or 100 mg of *P. infestans* mycelium was ground in liquid nitrogen (LN_2_), resuspended in 100 μl of 2× sodium dodecyl sulfate–polyacrylamide gel electrophoresis (SDS‐PAGE) sample loading buffer (100 mM Tris, 4% SDS, 20% glycerol, and 0.2% bromophenol blue) and loaded onto a 12% Bis‐Tris NuPAGE Novex gel (Invitrogen). The gel was run with 1× MES SDS running buffer (Invitrogen) for 30 min at 80 V, then at 110 V for another 1 h. Gel blotting onto nitrocellulose membrane, Ponceau staining, membrane blocking and washing steps were carried out as described by McLellan *et al*. (2013). αmRFP primary antibody (Sigma‐Aldrich) was used at 1 : 4000 dilution, while αGFP and αH3 antibodies (Sigma‐Aldrich) were used at 1 : 2000 dilution. Secondary antibodies anti‐rat immunoglobulin G (IgG) horseradish peroxidase (HRP) or anti‐rabbit IgG HRP (Sigma‐Aldrich) were used at 1 : 5000 dilutions. Protein bands on immunoblots were detected using ECL substrate (Thermo Scientific Pierce, Rockford, IL, USA) using the manufacturer's protocol.

## Results

### Cytoplasmic effector Pi04314 expressed in the host does not re‐enter plant cells after secretion

**Figure 1:**
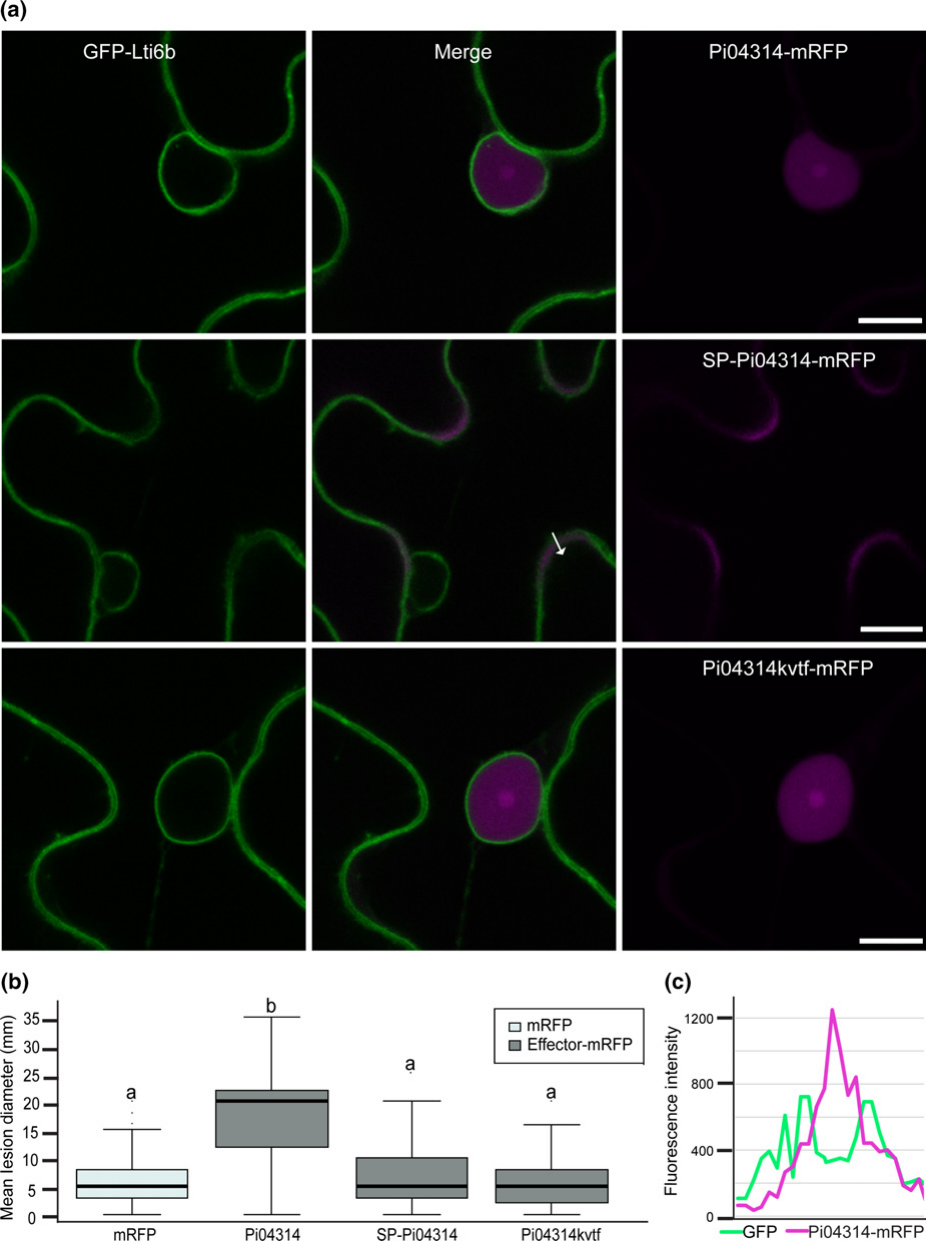
The secreted cytoplasmic effector *Phytophthora infestans* 04314 (Pi04314) does not show pathogen‐independent uptake into host cells. (a) Images are single optical sections of C‐terminal monomeric red fluorescent protein (mRFP)‐tagged Pi04314 (top panels), signal peptide (SP)‐Pi04314 (middle panels) and Pi04314kvtf mutant (bottom panels) fusion proteins expressed in transgenic *Nicotiana benthamiana* in which the plasma membrane and nuclear membrane were labelled with GFP‐LTi6b. The effector fusions are localized to the nucleus and nucleolus, when expressed without an SP. With an SP (SP‐Pi04314‐mRFP), the effector fusion is secreted from the plant cell into the apoplast and is not observed inside the cells. The arrow indicates the path used for the fluorescence intensity profile in (c). Bars, 10 μm. (b) *Phytophthora infestans* colonization of *N. benthamiana* significantly increased following *Agrobacterium tumefaciens*‐mediated expression of Pi04314‐mRFP compared with free mRFP, but no enhanced colonization was observed following expression of SP‐Pi04314‐mRFP or Pi04314kvtf‐mRFP. Error bars are ± SE, and the graph represents the combined data from three biological replicates (*n *=* *108 per construct). Different letters on the graph denote statistically significant differences (ANOVA;* P *<* *0.001). (c) A profile of the fluorescence intensities of both fluorophores across the plasma membranes and apoplast of adjoining cells as indicated in (a).

**Figure 2:**
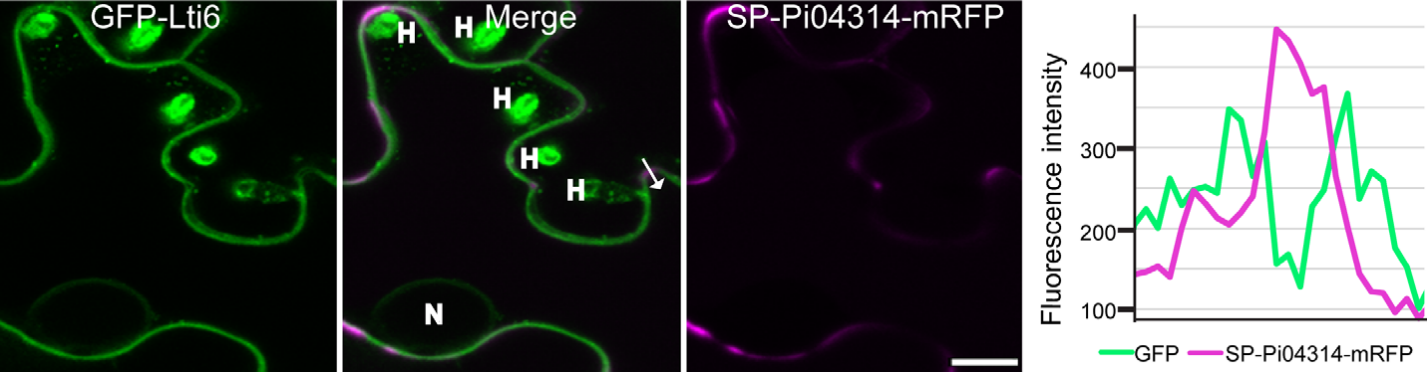
Secreted *Phytophthora infestans* 04314 (Pi04314) does not re‐enter the plant cell. *Phytophthora infestans* infection is shown on plants stably expressing the GFP‐LTi6b plasma membrane marker and transiently expressing secreted Pi04314‐monomeric red fluorescent protein (mRFP). The effector fusion did not re‐enter the plant cells from where it was secreted even in the presence of haustoria. The white arrow indicates the line used for the fluorescence intensity profile across the plasma membranes and the apoplast of adjoining cells shown in the graph to the right of the image. H, haustorium; N, nucleus. Bar, 10 μm. This is a representative image from three independent biological replicate experiments.

Previous studies of the function and localization of the RXLR effector Pi04314 utilized N‐terminal fluorescent protein fusions (Boevink *et al*., 2016). To study the translocation of Pi04314 from *P. infestans,* a C‐terminal mRFP fusion was required to avoid interfering with the N‐terminal secretion and delivery domains of the effector. As expected, Pi04314‐mRFP expressed transiently in *N. benthamiana* without a signal peptide (SP) accumulated in the nucleus and nucleolus (Figs 1a, S1) and re‐localized PP1c from the nucleolus (Fig. S2). By contrast, a KVTF mutant of the effector, as anticipated (Boevink *et al*., 2016), still accumulated in the nucleolus but failed to re‐localize PP1c (Figs 1a, S2). Moreover, Pi04314‐mRFP expression enhanced *P. infestans* colonization, whereas the KVTF mutant failed to do this (Fig. 1b), indicating that the C‐terminal mRFP fusion to wild‐type Pi04314 did not prevent effector activity. Interestingly, expression of SP‐Pi04314‐mRFP to secrete the effector from the plant cell revealed that it did not passively re‐enter the cell upon secretion, as no accumulation of mRFP was observed in the nucleolus (Fig. 1a), and instead the effector accumulated only in the apoplastic space (Fig. 1c). Moreover, SP‐Pi04314‐mRFP expression failed to enhance *P. infestans* colonization (Fig. 1b), suggesting that the effector does not re‐enter plant cells even when pathogen haustoria are present. Indeed, this was the case; secreted Pi04314‐mRFP remained in the apoplast of cells that were in intimate contact with haustoria (Fig. 2). Thus, our assay, involving both visualization of secretion and pathogen challenge, does not support re‐entry of a secreted cytoplasmic effector into the host cell, even in the presence of the pathogen.

### *Phytophthora infestans* delivers Pi04314‐mRFP into the host nucleus from haustoria

**Figure 3:**
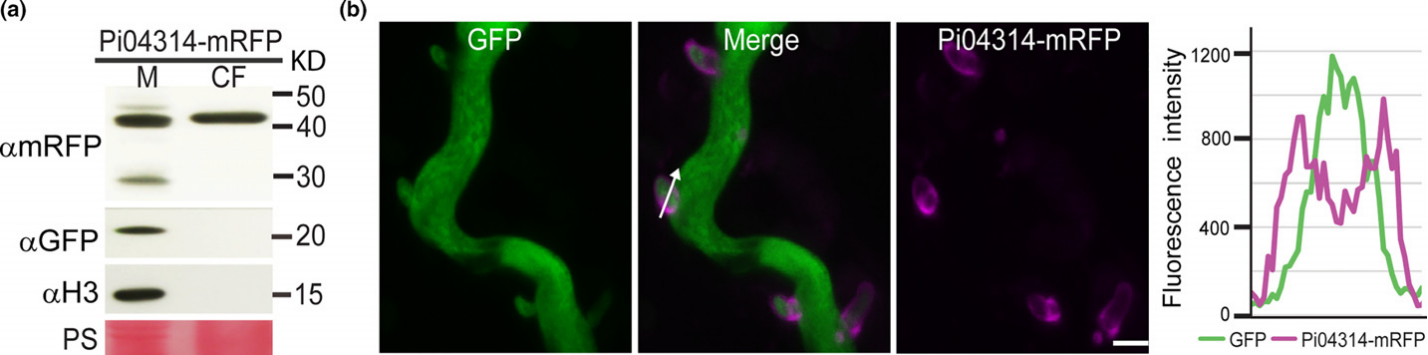
*Phytophthora infestans* 04314 (Pi04314) is secreted from mycelium into the culture filtrate (CF) and from haustoria *in planta*. (a) Expression of the Pi04314‐monomeric red fluorescent protein (mRFP) fusion protein in the mycelium (M) and into the CF of *in vitro*‐grown *Phytophthora infestans* was confirmed using an mRFP antibody. Green fluorescent protein (αGFP) and histone H3 (αH3) antibodies were used as indicators of mycelial proteins and showed that the CF was not detectably contaminated with these cellular markers. Size markers are indicated in kDa, and protein loading is indicated by Ponceau stain (PS). This immunoblot is representative of three independent biological replicates. (b) Projection of a confocal z‐series of *P. infestans* transformant 1 expressing eGFP in the hyphal cytoplasm and Pi04314‐mRFP. Secretion of Pi04314 was observed to occur at haustoria. The white arrow shows the line used for the fluorescence intensity profile indicated in the graph to the right of the images. Bar, 5 μm. These images are representative of three independent biological replicates, each resulting in > 10 images of haustoria on independent infected leaves.

To observe whether the pathogen could deliver Pi04314 to its site of activity in the host cell, we generated six *P. infestans* transformants expressing SP‐Pi04314‐mRFP under the control of the constitutive Ham34 promoter. The vector used also allowed constitutive expression of free eGFP in the pathogen cytoplasm (Fig. S3a). The expression of SP‐Pi04314‐mRFP was investigated in two independent *in vitro*‐grown *P. infestans* transformants (Figs 3a, S3b), demonstrating that the intact effector‐mRFP fusion was secreted into the CF in each case. By contrast, histone H3 and cytoplasmic eGFP were detected only in the mycelium, indicating little or no contamination of the CF with intracellular proteins. Protein secretion from cultured *P. infestans* hyphae without haustoria is well documented (Torto *et al*., 2003; Meijer *et al*., 2014). However, it was not possible here to observe the sites of secretion *in vitro* using confocal microscopy, as the fusion protein would be rapidly diluted upon secretion. In contrast to *P. infestans* expressing Pi04314‐mRFP, transgenic pathogen expressing free mRFP alone showed that the fluorescent protein was only detected in the mycelium and was not secreted into the CF (Fig. S4a).

During infection, mRFP fluorescence was detected at the neck of the haustorium and in the extra‐haustorial matrix (EHMx) for each of the independent SP‐Pi04314‐mRFP transformants (Figs 3b, S3c). This is in agreement with previous studies of effectors Avr3a (Whisson *et al*., 2007, 2016) and Avr2 (Gilroy *et al*., 2011) from *P. infestans*, and Avr1b (Liu *et al*., 2014) from *Phytophthora sojae*, which suggest the haustorium as a site for RXLR effector delivery. By contrast, fluorescence from transformed *P. infestans* expressing only free mRFP indicated that mRFP fluorescence was detected throughout the mycelium and did not specifically accumulate at haustoria (Fig. S4b).

**Figure 4:**
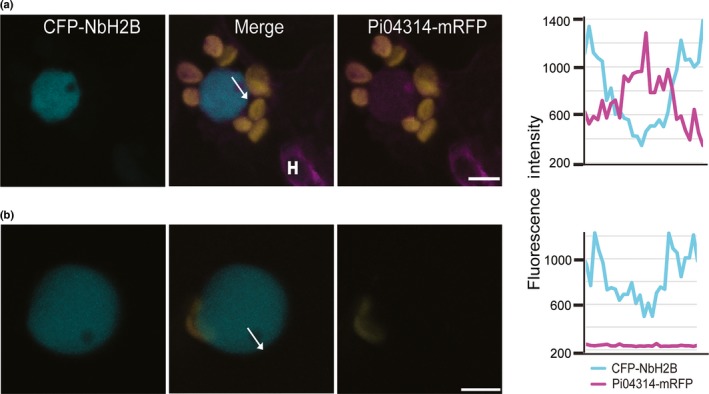
The *Phytophthora infestans* 04314−monomeric red fluorescent protein (Pi04314‐mRFP) fusion protein is translocated from the pathogen into the host nucleus and nucleolus. (a) Representative confocal projections of a nucleus near a haustorium (H) in an infected cell in which the nucleus was labelled by CFP‐*Nb*H2B. The nucleolus and nucleoplasm contain red fluorescence, indicating that Pi04314 fusion proteins have translocated from haustoria to the host nucleus and nucleolus. Chloroplast autofluorescence is indicated in yellow. The white arrow shows the line used for the fluorescence intensity profile indicated in the graph to the right of the images. These images are representative of 31 independent images of haustoriated cells from four independent biological replicates, from two independent transformants. (b) A single optical section of a typical nucleus in a nonhaustoriated cell from an infected leaf; no red fluorescence can be observed in the nucleus. Bars, 5 μm.

Importantly, for each of the independent SP‐Pi04314‐mRFP transformants, mRFP fluorescence was also detected in the host nucleus, where it strongly accumulated in the nucleolus (Figs 4a, S3d), providing the first direct observation of effector translocation by an oomycete plant pathogen. From four independent replicated experiments for each transformant, a total of 76 haustoriated plant cells were studied using confocal microscopy. Of the 76 haustoriated host cells, clear mRFP fluorescence was detected in the nuclei in 31 cases. By contrast, no mRFP fluorescence was detected in hundreds of neighbouring, nonhaustoriated host cells (example shown in Fig. 4b).

In conclusion, SP‐Pi04314‐mRFP expressed in *P. infestans* transformants was secreted *in vitro*, and *in planta* led to accumulation of mRFP fluorescence at the haustorium and in the host nucleoplasm and nucleolus. By contrast, *A. tumefaciens‐*mediated 35S‐driven expression of SP‐Pi04314‐mRFP in host cells led to secretion of the effector into the apoplast, but no re‐entry into the plant cell (Figs 1, 2). This distinction calls into question whether Pi04314‐mRFP is conventionally secreted by *P. infestans* into the EHMx as, when Pi04314‐mRFP is secreted from the plant, even the presence of pathogen haustoria did not result in effector re‐entry into host cells.

### *Phytophthora infestans* apoplastic effector EPIC1 is also secreted from haustoria

**Figure 5:**
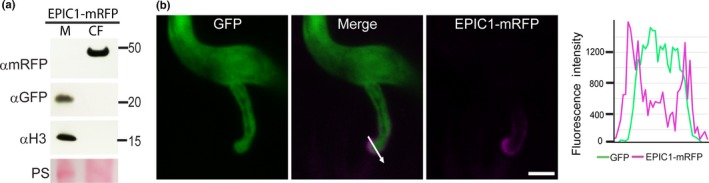
The apoplastic effector EPIC1 is secreted from haustoria. (a) Expression of the EPIC1‐monomeric red fluorescent protein (mRFP) fusion protein in *in vitro*‐grown *Phytophthora infestans* was confirmed using an mRFP antibody; the protein was secreted into the filtered culture medium (CF). Green fluorescent protein (αGFP) and histone H3 (αH3) antibodies were used as indicators of cellular proteins as described for the *Phytophthora infestans* 04314 (Pi04314) transformant in Fig. 3. Markers are indicated in kDa, and protein loading is indicated by Ponceau stain (PS). M, mycelial fraction. (b) Confocal projection of a section of hyphae and a haustorium of the EPIC1‐mRFP transformant infecting *Nicotiana benthamiana* leaf tissue, showing that the EPIC1 fusion is secreted at haustoria. The white arrow indicates the line used for the fluorescence intensity profile shown in the graph to the right of the images. Bar, 5 μm.

We selected the effector EPIC1, which is known to interact with and inhibit secreted plant defence proteases in the apoplast (Song *et al*., 2009), to provide a contrast to the cytoplasmic effector Pi04314. EPIC1 with its signal peptide for secretion was C‐terminally tagged with mRFP for expression in *P. infestans*, with free eGFP expressed to label *P. infestans* hyphae (Fig. S5a). In two independent transformants, fusion‐protein expression was confirmed by immunoblot, showing that mature EPIC1‐mRFP fusion protein was secreted into the CF when grown *in vitro* (Figs 5a, S5b). During infection of *N. benthamiana*, confocal projections showed that mRFP fluorescence accumulated strongly around the haustorium (Figs 5b, S5c). Thus, the haustorium is a site for secretion not only of the cytoplasmic RXLR effectors but also of the apoplastic effector EPIC1.

### Pi04314 and EPIC1 are secreted by distinct secretory pathways

**Figure 6:**
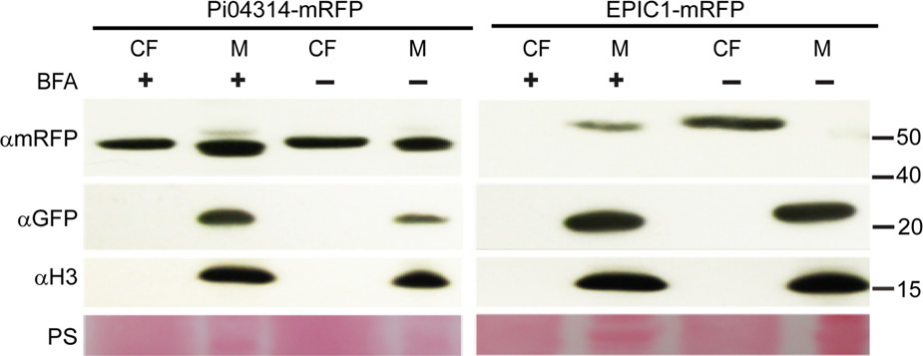
Brefeldin A (BFA) inhibits secretion of an apoplastic effector but not a cytoplasmic effector *in vitro*. The effect of BFA treatment was assayed by immunoblotting which showed there was no effect on the secretion of the cytoplasmic effector *Phytophthora infestans* 04314 (Pi04314) after 3 h of BFA treatment, while the secretion of the apoplastic effector EPIC1 was blocked. αmRFP, αGFP and αH3 were used as primary antibodies to detect monomeric red fluorescent protein (mRFP), green fluorescent protein (GFP) and histone H3, respectively. αGFP and αH3 were used as indicators of cellular protein to show there was no contamination of the filtered culture medium (CF) by mycelial (M) proteins. Samples from transformant cultures treated with BFA are indicated by +. Protein size markers are indicated in kDa and protein loading is indicated by Ponceau stain (PS). These immunoblots are representative of three independent biological replicates (Supporting Information Fig. S6) showing similar results.

Given that Pi04314 and EPIC1 have been shown to function inside and outside of host cells, respectively, we investigated the process of secretion from the pathogen in each case. *Phytophthora infestans* transformants expressing SP‐Pi04314‐mRFP or EPIC1‐mRFP, grown *in vitro*, were exposed to BFA, which inhibits conventional endoplasmic reticulum (ER)‐to‐Golgi secretion (Chardin & McCormick, 1999). Secretion of apoplastic effector PiEPIC1‐mRFP was inhibited by BFA treatment, with fusion protein detected solely in the mycelium fraction rather than the CF (Figs 6, S6). In contrast, the same BFA treatment had little or no effect on the secretion of the cytoplasmic effector Pi04314‐mRFP (Figs 6, S6).

**Figure 7:**
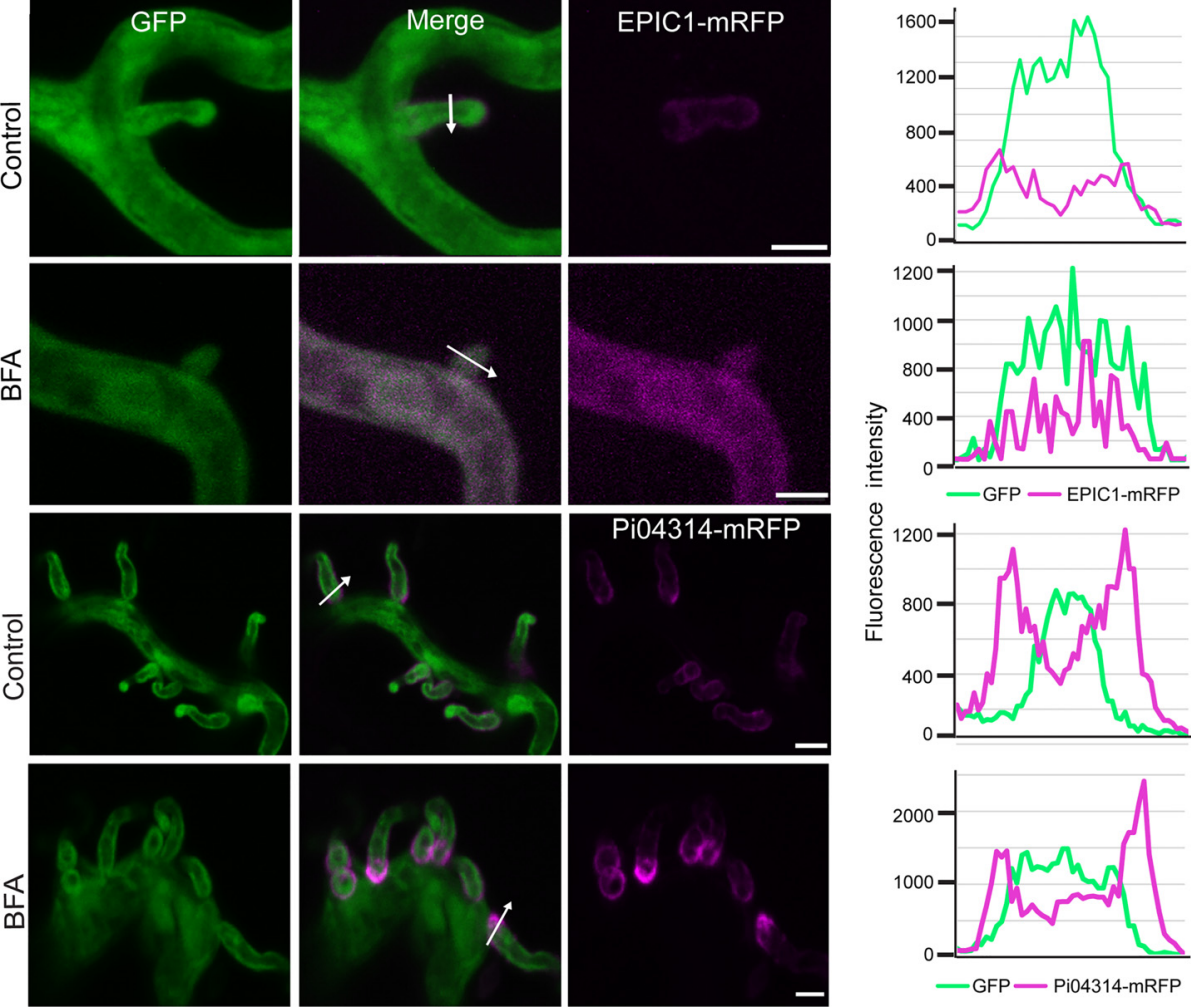
Brefeldin A (BFA) blocks secretion of an apoplastic effector but not a cytoplasmic effector during infection. Confocal projections of the same transformants in infected *Nicotiana benthamiana* reveal that the cytoplasmic effector *Phytophthora infestans* 04314−monomeric red fluorescent protein (Pi04314‐mRFP) is preferentially secreted from haustoria even in the presence of BFA, but the apoplastic effector EPIC1 is retained in the hyphae and haustoria rather than outlining haustoria as in the water treatment control. White arrows indicate the lines used for the fluorescence intensity profiles shown in graphs to the right of each image. The BFA panel was imaged after 3 h of exposure to BFA. Bars, 5 μm. These images are representative of three independent biological replicates.

In agreement with observations following *in vitro* growth, confocal microscopy revealed that mRFP fluorescence from *P. infestans* expressing the apoplastic effector EPIC1‐mRFP accumulated inside hyphae and haustoria following 3 h of exposure of infected *N. benthamiana* leaves to BFA, suggesting that secretion had been inhibited (Fig. 7). By contrast, secretion of Pi04314‐mRFP from *P. infestans* was insensitive to BFA treatment, as mRFP fluorescence accumulated around haustoria, with no accumulation of fluorescence inside the hyphae (Fig. 7), even following longer periods of exposure to BFA. This indicates that the accumulation of fluorescence specifically in hyphae of transformants expressing EPIC1‐mRFP is attributable to the fusion protein not being secreted, rather than autofluorescence generated as a prelude to death following BFA treatment.

**Figure 8:**
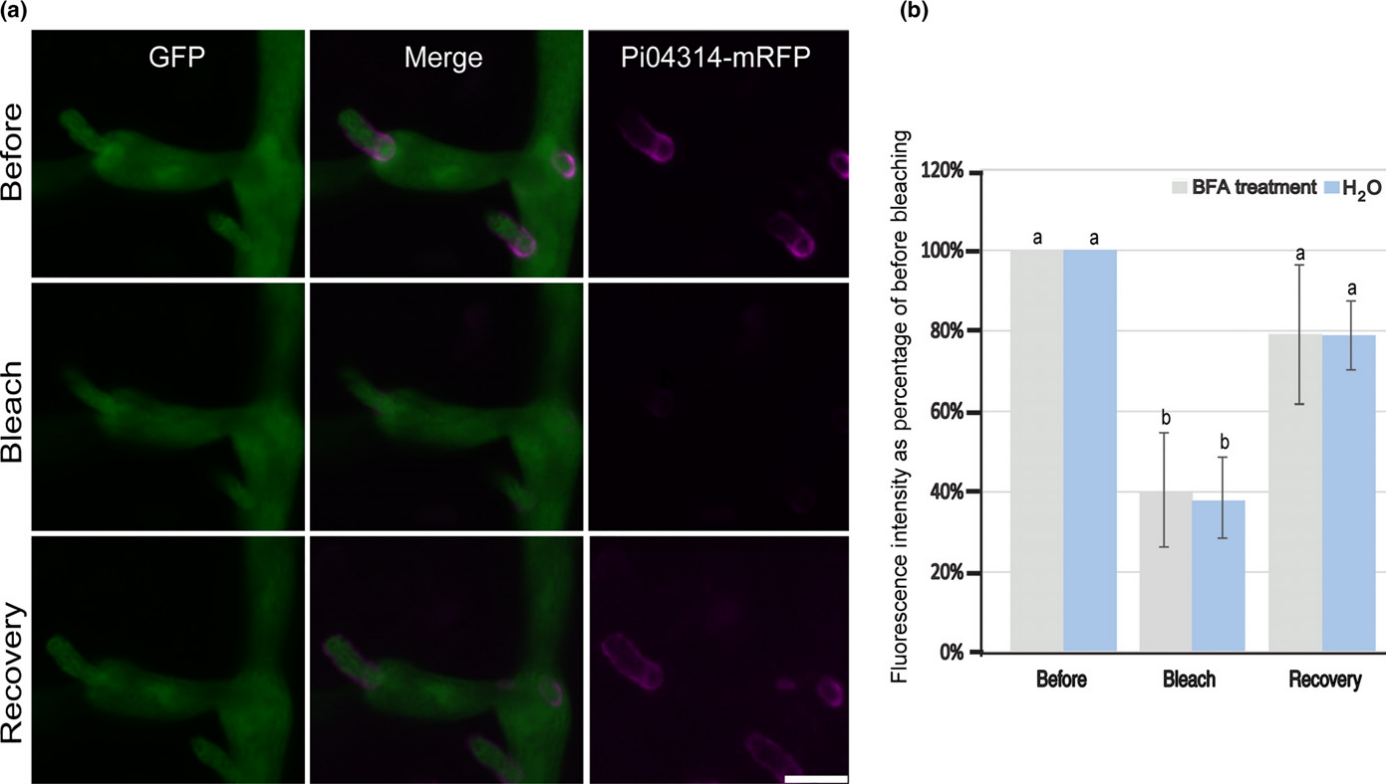
Fluorescence recovery after photobleaching (FRAP) confirms that *Phytophthora infestans* 04314 (Pi04314) is secreted by a nonconventional secretion pathway during infection. (a) Images of example haustoria pre‐bleach (before), immediately post‐bleach (bleach) and after 3 h of recovery. Bar, 5 μm. (b) FRAP results from eight bleached haustoria were identical in the presence or absence of brefeldin A (BFA) (*P *<* *0.01), demonstrating continued secretion of Pi04314‐monomeric red fluorescent protein (mRFP) in the presence of BFA. Bars show mean red fluorescence intensity collected before bleaching, immediately after bleaching and after 3 h of recovery time. Error bars are ± SE, the pre‐bleach fluorescence intensity of Pi04314‐mRFP was normalized at 100%, and different lowercase letters denote statistical difference (*P *<* *0.01) using one‐way ANOVA.

To further investigate the BFA‐insensitive secretion of Pi04314‐mRFP, we performed FRAP. Using confocal microscopy, mRFP fluorescence was photobleached around haustoria and fluorescence recovery was monitored over time in the presence of BFA or, as a control, water treatment. Full recovery of mRFP fluorescence was observed around haustoria within 3 h following water treatment. A significantly similar recovery rate was observed with BFA treatment (Fig. 8).

## Discussion

In this study, we visualized the delivery of a cytoplasmic oomycete RXLR effector to its site of action in the host nucleus. Both the cytoplasmic effector Pi04314 and an apoplastic effector, EPIC1, were secreted from haustoria, demonstrating the importance of these structures during infection. However, whereas EPIC1 was delivered by conventional ER‐to‐Golgi secretion, Pi04314 delivery was not dependent on Golgi‐mediated secretion. Each of these points is discussed in turn below.

Initially, we tested whether C‐terminal mRFP fusion to cytoplasmic effector Pi04314 remained functional in plant cells, as such fusion is required to avoid interfering with the predicted N‐terminal translocation domain of the effector. Indeed, expression of Pi04314‐mRFP inside plant cells without a secretion SP resulted in effector accumulation in the host nucleolus, re‐localization of PP1c from the nucleolus, and enhanced *P. infestans* colonization of *N. benthamiana* as shown for N‐terminal fluorescent protein fusions (Boevink *et al*., 2016). Thus, fusion of mRFP to the C‐terminus of Pi04314 did not attenuate its function. However, expression of SP‐Pi04314‐mRFP transiently in *N. benthamiana*, although clearly leading to secretion of the effector and its accumulation in the apoplast, failed to result in effector re‐entry into the cell or enhanced *P. infestans* colonization. Pathogen‐independent uptake of cytoplasmic effectors has been a hotly debated topic; ‘cell‐re‐entry assays’ (following effector secretion from the plant cell) and ‘uptake assays’ (involving direct application of recombinant effector‐fluorescent protein fusions to plant tissues) have provided conflicting results (Tyler *et al*., 2013; Wawra *et al*., 2013; Petre & Kamoun, 2014). In most reported cases, however, visualization of secretion, or of appropriate effector (re)uptake to reach the site of activity in the plant cell, has not been demonstrated. Our assays do not support pathogen‐independent uptake of the effector Pi04314.

To date, the sites of secretion of oomycete apoplastic effectors during infection have not been explored. The strong accumulation of EPIC1‐mRFP in the EHMx indicates that apoplastic effectors may be secreted from haustoria. Moreover, it suggests that host proteases such as tomato apoplastic cysteine protease (Rcr3), papain‐like cysteine protease (C14) and Phytophthora Inhibited Protease (PIP1), which are targets for inhibition by EPIC1 (Tian *et al*., 2007; Song *et al*., 2009; Kaschani *et al*., 2010), are secreted by the host at the extrahaustorial membrane (EHM). Indeed, consistent with this, the *P. infestans* RXLR effector AvrBlb2 has been shown to hyper‐accumulate at the EHM and reported to block secretion of C14 when transiently expressed inside host cells (Bozkurt *et al*., 2011). Secretion of EPIC1 at haustoria is thus logical to battle defence proteases secreted by the plant at this site. In addition, an analogous cysteine protease effector Pit2 from *U. maydis*, was shown to be secreted and to accumulate in the biotrophic interface surrounding intercellular hyphae, a location that is also in intimate association with host cells (Mueller *et al*., 2013).

Following growth of transformants either *in vitro* or *in planta*, EPIC1‐mRFP secretion was observed to be inhibited by BFA, whereas Pi04314‐mRFP was insensitive to BFA, revealing that these two effectors follow different secretion pathways. This is in agreement with a study of the fungal rice blast pathogen *M. oryzae*, showing that delivery of an apoplastic effector followed conventional, BFA‐sensitive secretion, whereas cytoplasmic effectors were translocated into the host cell in a BFA‐insensitive manner (Giraldo *et al*., 2013). Future work will reveal whether this is a general rule for fungal and oomycete plant pathogens, and whether the nonconventional secretion and translocation of cytoplasmic effectors follow similar principles in distantly related filamentous pathogens.

In contrast to oomycete apoplastic effectors, a number of studies have shown that RXLR effector‐mRFP fusion proteins accumulate at haustoria (Whisson *et al*., 2007, 2016; Gilroy *et al*., 2011; Liu *et al*., 2014), implicating this as a site of their delivery. However, in none of these cases was the effector fusion protein shown to accumulate inside the plant cell at its subcellular site of activity. Pi04314‐mRFP accumulation in the host nucleoplasm and nucleolus is consistent with its activity in forming holoenzymes with plant PP1c isoforms in the nucleus (Boevink *et al*., 2016) and is thus the first visual demonstration of RXLR effector translocation. The secretion of both apoplastic and cytoplasmic effectors from haustoria emphasizes the importance of the haustorial interface as a battleground between pathogen and host. Future work studying a wider range of secreted proteins can determine the extent to which the haustorium is a major, or general, site of secretion during infection. The observed delivery of Pi04314‐mRFP inside the plant nucleus provides an exciting opportunity to study the translocation process and the role of the RXLR motif in the nonconventional secretion of this effector from the pathogen cell to the inside of the plant cell.

## Author contributions

S.W., P.C.B., R.Z., S.C.W. and P.R.J.B. designed the research. S.W., P.C.B. and L.W. performed the research. S.W., P.C.B., S.C.W. and P.R.J.B. analysed the data. S.W. and P.R.J.B. wrote the article with contributions and comments from all authors.

## Supporting information

Please note: Wiley Blackwell are not responsible for the content or functionality of any Supporting Information supplied by the authors. Any queries (other than missing material) should be directed to the *New Phytologist* Central Office.

**Fig. S1** Transient *in planta* expression assays for Pi04314‐mRFP.
**Fig. S2** C‐terminally tagged wild‐type Pi04314 re‐localizes GFP‐PP1c from the nucleolus.
**Fig. S3** Immunoblotting and images show that an additional transformant expressing the Pi04314‐mRFP fusion gives consistent results.
**Fig. S4** *Phytophthora infestans* transformants expressing free mRFP show that it is not secreted and does not specifically accumulate at haustoria.
**Fig. S5** Secretion of apoplastic effector EPIC1 from an independent *Phytophthora infestans* transformant.
**Fig. S6** In independent biological replicates (REPs), BFA treatment (+) inhibits secretion of EPIC1‐mRFP into the culture filtrate (CF) but has little or no inhibitory effect on Pi04314‐mRFP secretion.
**Table S1** Oligonucleotide primers used in Pi04314 plasmid construction
