# Supplementary material for: Delivery of cytoplasmic and apoplastic effectors from Phytophthora infestans haustoria by distinct secretion pathways
Source: New Phytol. 2017 Jul 31;216(1):205–15. doi: 10.1111/nph.14696 (PMC5601276; doi:10.1111/nph.14696)
Supplement: Supplementary file 1 — Fig. S1 Transient in planta expression assays for Pi04314‐mRFP. Fig. S2 C‐terminally tagged wild‐type Pi04314 re‐localizes GFP‐PP1c from the nucleolus. Fig. S3 Immunoblotting and images show that an additional transformant expressing the Pi04314‐mRFP fusion gives consistent results. Fig. S4 Phytophthora infestans transformants expressing free mRFP show that it is not secreted and does not specifically accumulate at haustoria. Fig. S5 Secretion of apoplastic effector EPIC1 from an independent Phytophthora infestans transformant. Fig. S6 In independent biological replicates (REPs), BFA treatment (+) inhibits secretion of EPIC1‐mRFP into the culture filtrate (CF) but has little or no inhibitory effect on Pi04314‐mRFP secretion. Table S1 Oligonucleotide primers used in Pi04314 plasmid construction [file NPH-216-205-s001.pdf]

Supporting Information

Delivery of cytoplasmic and apoplastic effectors from *Phytophthora infestans* haustoria by distinct secretion pathways

Shumei Wang[1], Petra C Boevink[2], Lydia Welsh[2], Ruofang Zhang[3], Stephen C Whisson[2], Paul RJ Birch[1,2]*

[1]Division of Plant Sciences, University of Dundee (at JHI), Errol Road, Invergowrie, Dundee DD2 5DA, UK

[2]Cell and Molecular Sciences, James Hutton Institute, Errol Road, Invergowrie, Dundee DD2 5DA, UK.

[3] Potato Engineering and Technology Research Centre of Inner Mongolia University, West College Road 235, Hohhot, 010021, China.

*Author for correspondence: Paul.Birch@hutton.ac.uk

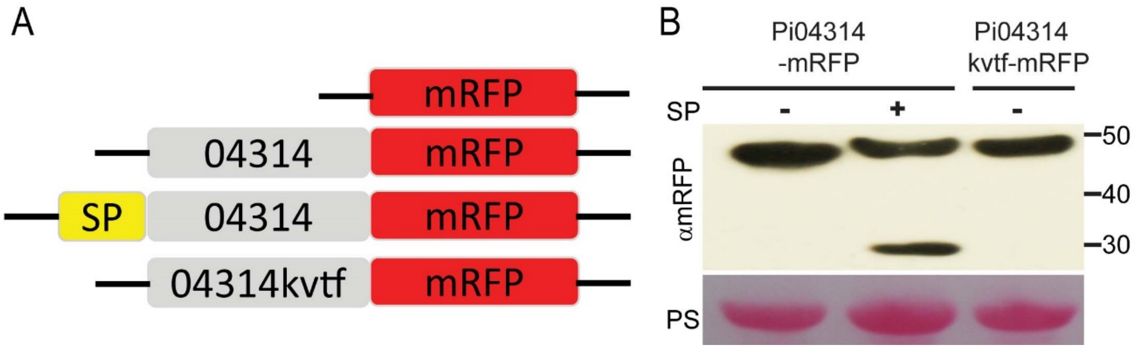

Fig. S1. Transient *in planta* expression assays for Pi04314-mRFP. A. Diagrams of the four constructs: free mRFP, Pi04314 with or without signal peptide (SP) fused to mRFP, and Pi04314 kvtf mutant fused to mRFP. **B.** Immunoblotting with an αmRFP primary antibody shows that Pi04314-mRFP (+/- SP) and Pi04314kvtf-mRFP fusion proteins are stable when expressed in plant cells. Construct with SP is indicated by +. Protein size markers are indicated in kDa and Ponceau stain (PS) indicates protein loading.

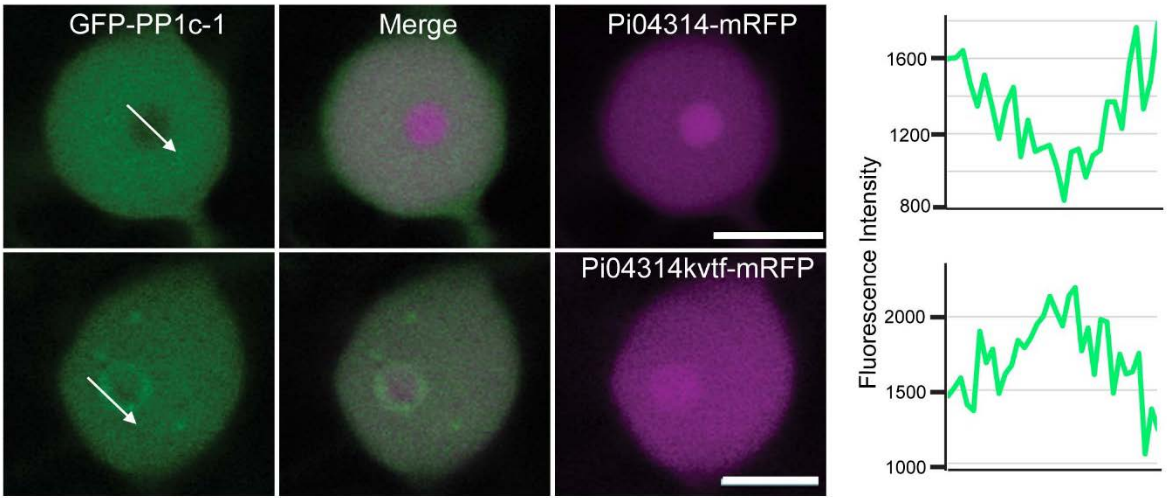

Fig. S2. C-terminally tagged wild-type Pi04314 re-localises GFP-PP1c from the nucleolus. Projections of nuclei co-expressing Pi04314-mRFP or Pi04314kvtf-mRFP with GFP-PP1c in *N. benthamiana* cells confirm that the wild-type effector form re-localises PP1c with a C-terminal fluorescent protein fusion. As expected, the mutant form of the effector was non-functional, in that it failed to re-localise GFP-PP1c from the nucleolus. White arrows indicate the lines used for GFP fluorescence intensity profiles shown in the graphs to the right of each image set. Scale bar = 5 µm.

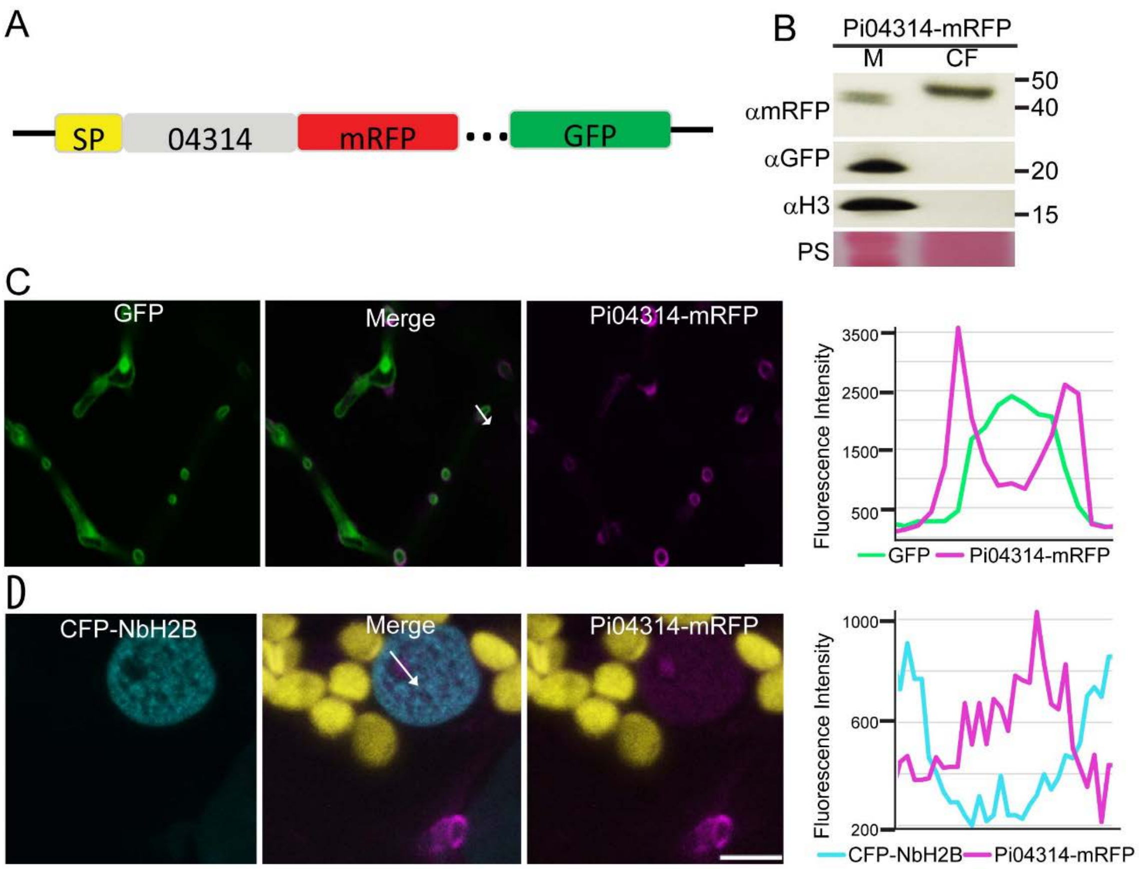

Fig. S3. Immunoblotting and images show that an additional transformant expressing the Pi04314-mRFP fusion gives consistent results. A. A diagram of the construct for transformation of *P. infestans* to express the Pi04314-mRFP fusion with signal peptide (SP) for secretion. GFP for labelling the hyphae was expressed from a separate promoter on the same plasmid. **B.** Immunoblotting was performed to show the expression of Pi04314-mRFP fusion proteins in the transformant with mRFP primary antibody. αGFP and αH3 were used as before (Figure 2) to indicate the presence of cellular proteins only in the mycelial sample. Protein size markers are indicated in kDa and protein loading was confirmed by Ponceau stain (PS). M= mycelium, CF= culture filtrate. **C.** Confocal projection of the same transformant used in (B), confirming the haustorial location of Pi04314-mRFP secretion. Scale bar represents 5 µm. **D.** Confocal projections of a nucleus in a haustoriated cell, confirming Pi04314-mRFP translocation from the transformed pathogen into host cells is a consistent behaviour. White arrows indicate the lines used for the fluorescence intensity profiles shown in the graphs to the right of each image. H=haustorium. Scale bar represents 5 µm.

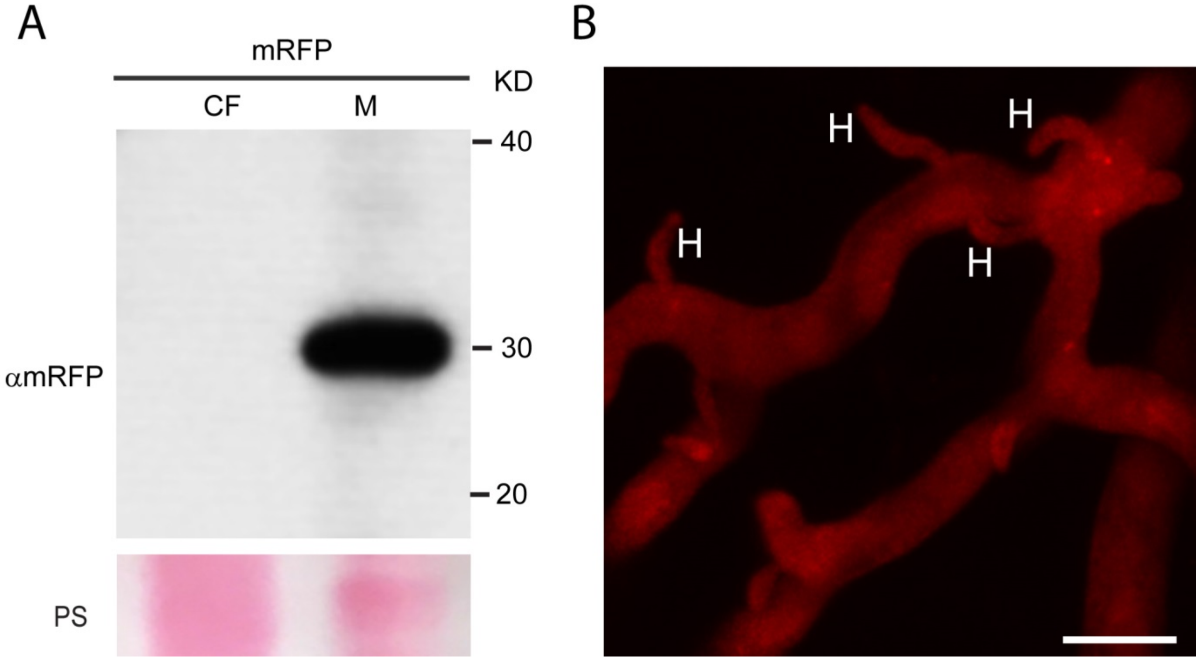

Fig. S4. ***Phytophthora infestans*** **transformants expressing free mRFP show that it is not secreted and does not specifically accumulate at haustoria. A.** Free mRFP expressed in *P. infestans* is detectable in the mycelium (M) but not the culture filtrate (CF) following growth *in vitro*. PS is Ponceau stain and kD is size in kiloDaltons. **B**. Transgenic *P. infestans* expressing free mRFP shows that it does not specifically accumulate at haustoria (H). Scale bar is 10 µm.

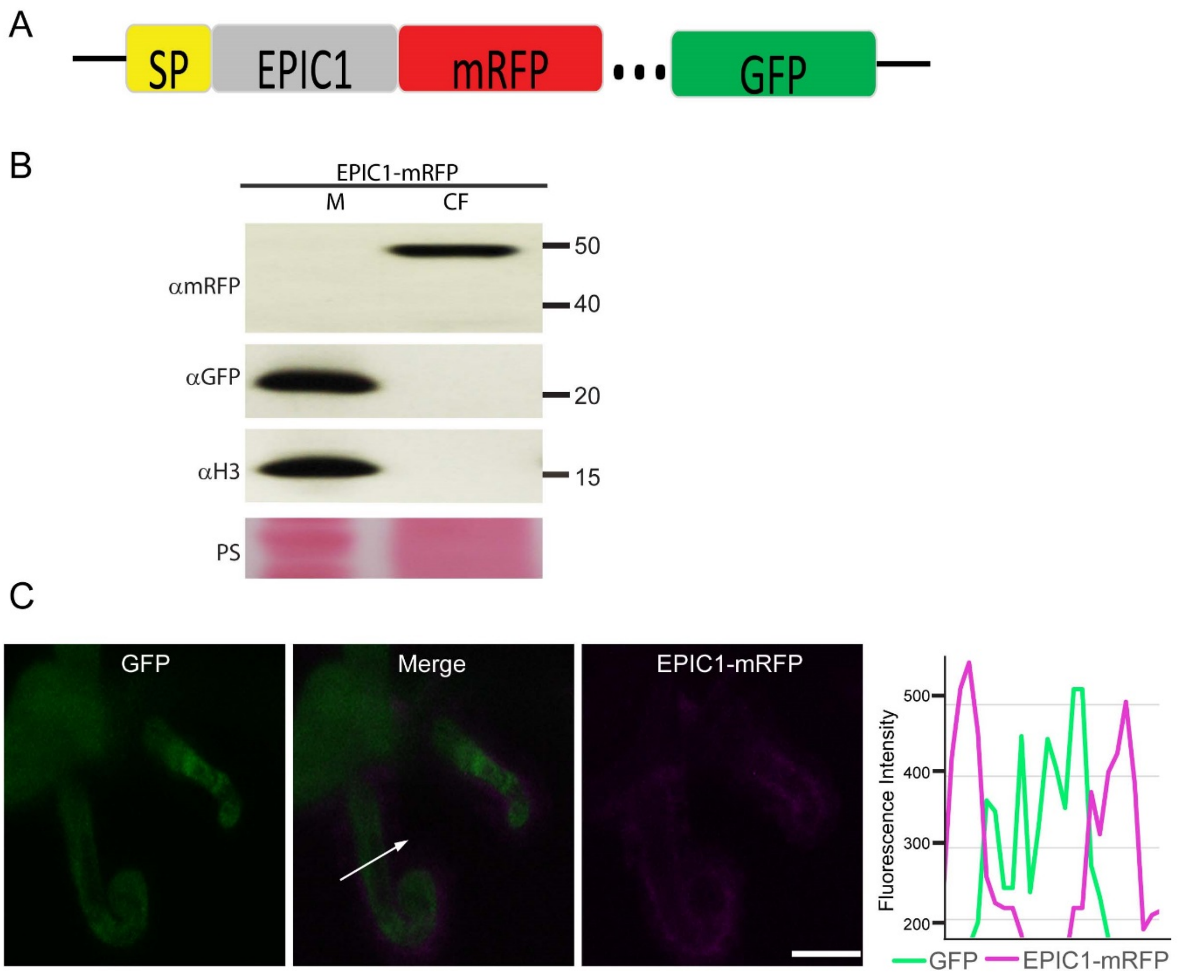

Fig. S5. Secretion of apoplastic effector EPIC1 from an independent *P. infestans* transformant. A. A diagram of the construct for *P. infestans* transformation to express EPIC1-mRFP with a signal peptide (SP) and GFP for labelling hyphae from a separate promoter. **B.** The expression and secretion of the fusion protein from the *in vitro*-grown *P. infestans* transformant was confirmed by immunoblotting mycelia (M) and filtered culture medium (CF) with αmRFP primary antibody. αGFP and αH3 antibodies were used as markers to indicate cellular proteins. Protein size markers are indicated in kDa and protein loading was confirmed by Ponceau stain (PS). **C.** A single optical section through a section of transformant hyphae with haustoria in infected *N. benthamiana* shows secretion of the apoplastic effector from haustoria. The white arrow indicates the line used for the fluorescence intensity profile shown in the graph to the right of the images. Scale bar is 10µm.

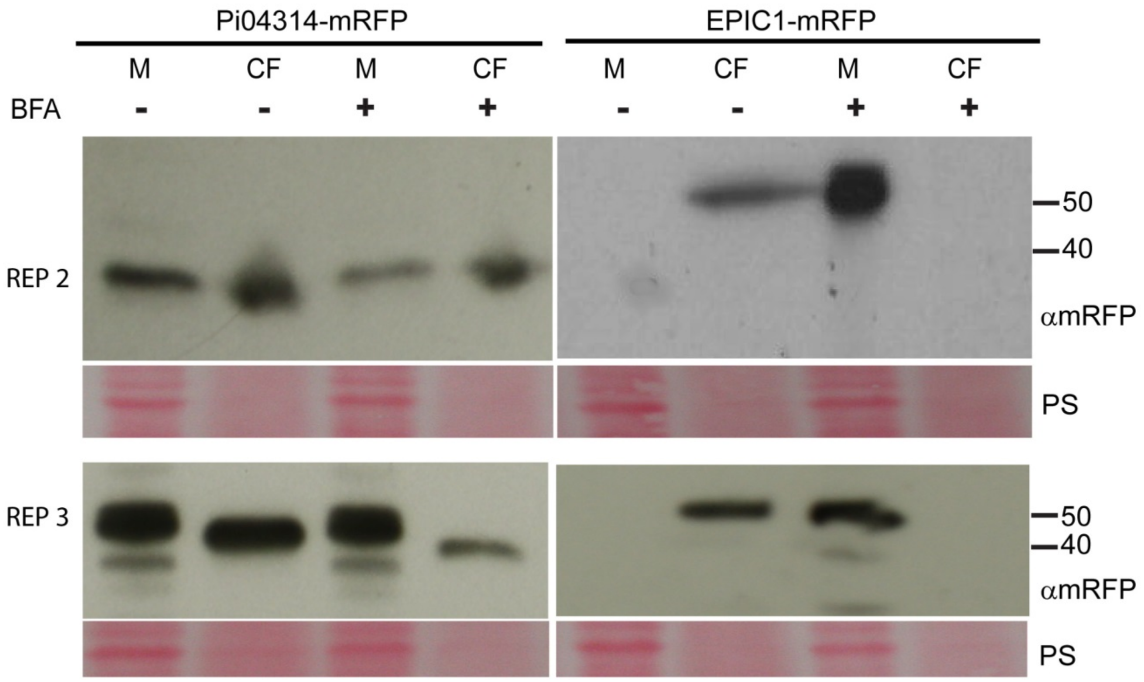

Fig. S6. In independent biological replicates (REP), BFA treatment (+) inhibits secretion of EPIC1-mRFP into the culture filtrate (CF) but has little or no inhibitory effect on Pi04314-mRFP secretion.

Table S1: Oligonucleotide primers used in Pi04314 plasmid construction

Primer name	Primer sequence
04314ClaI-F	GGAAATCGATACC ATGCATTCAAGTCTTCTTTGGTTAG
04314AsisIkpn1-R	GGAAGCGATCGCGGTACCGCCGAGTTGGTTTTGTAGAT ACGAGC
04314gateway-F1	AAAGCAGGCTTCACCATGGTATCGACCGAAGCTAAT
SP04314gateway-F1	AAAGCAGGCTTCACCATGCATTCAAGTCTTCTTTGGT
04314KVTFgateway-R	GGAAGGTACCGCCGAGTTGGTTTTGTAGATACGAGCTC GGACTTTGTTCGAGGCAGCCGCCGCGTTGGGATTGTT
EPIC1AsisI-F	GGAA GCGATCGCACCATGACATTCCTTCGCCCCATC
EPIC1AsisIkpn1-R	GGAAGCGATCGCGGTACCGCCTTAACTGGGGTAATC GACGTCA
EPIC1gateway-F1	AAAGCAGGCTTCACCATGGGCGGATACTCGAAGAAG
mRFPgateway-R	GAAAGCTGGGTCTTA GGCGCCGGTGGAGTG
Ham34Kpn1-F	GGAAGGTACCGGGCCCATTATACC
Ham34Kpn1-R	GGAAGGTACCGCTAACATTCAAGCGAGCATGA
attB-F2	GGGGACAAGTTTGTACAAAAAAGCAGGCT
attB-R2	GGGGACCACTTTGTACAAGAAAGCTGGG

Red font indicates restriction enzyme recognition sites: *Cla*I ATCGAT, *Asis*I GCGATCGC, *Kpn*1 GGTACC
